# The Evolving Role of Taxanes in Combination With Cetuximab for the Treatment of Recurrent and/or Metastatic Squamous Cell Carcinoma of the Head and Neck: Evidence, Advantages, and Future Directions

**DOI:** 10.3389/fonc.2019.00668

**Published:** 2019-08-21

**Authors:** Joël Guigay, Makoto Tahara, Lisa Licitra, Ulrich Keilholz, Signe Friesland, Pauline Witzler, Ricard Mesía

**Affiliations:** ^1^Centre Antoine Lacassagne, Université Côte d'Azur, Nice, France; ^2^Department of Head and Neck Medical Oncology, National Cancer Center Hospital East, Tokyo, Japan; ^3^Fondazione IRCCS Istituto Nazionale Tumori, University of Milan, Milan, Italy; ^4^Charité Comprehensive Cancer Center, Berlin, Germany; ^5^Karolinska Institutet, Stockholm, Sweden; ^6^Merck KGaA, Darmstadt, Germany; ^7^Medical Oncology Department, Catalan Institute of Oncology, B-ARGO Group–Badalona, Barcelona, Spain

**Keywords:** cetuximab, docetaxel, paclitaxel, EXTREME, TPEx, B490, R/M SCCHN

## Abstract

The addition of cetuximab to platinum-based chemotherapy (cisplatin or carboplatin plus 5-fluorouracil [5-FU]), followed by maintenance cetuximab until disease progression (EXTREME), resulted in the first regimen to yield significantly improved survival outcomes in the first-line treatment of patients with recurrent and/or metastatic squamous cell carcinoma of the head and neck (R/M SCCHN) in over 30 years. Currently, the EXTREME regimen is a guideline-recommended treatment in the first-line R/M setting, and, therefore, it is used as a control arm in all new first-line, phase 3 immunotherapy trials. More recently, new checkpoint inhibitor approaches have emerged and are changing the treatment landscape for PD-L1–positive patients with R/M SCCHN. Additionally, alternative chemotherapy backbones in R/M SCCHN are continually investigated. Replacing 5-FU with a taxane in the EXTREME regimen seeks to take advantage of the potential immunogenic and proapoptotic synergy between cetuximab and docetaxel or paclitaxel. These cetuximab-, platinum-, and taxane-based treatments have demonstrated promising survival results and cytoreductive properties in single-arm studies. Thus, these combination treatments may be of importance to patients with high tumor burden and dangerous site involvements (e.g., causing bleeding, suffocation, dysphagia, or ulceration), in whom symptom relief is a key treatment goal. TPExtreme is the first large, randomized trial comparing a cetuximab, platinum, and taxane combination regimen with EXTREME. Currently, the substitution of 5-FU with a taxane is a feasible and clinically beneficial option for patients with contraindications to 5-FU. The TPEx regimen appears to be a new option in first-line R/M SCCHN, with a shorter time on CT and significantly lower toxicity than the EXTREME regimen. For patients with R/M disease in whom further cisplatin- or carboplatin-based treatment is unsuitable, or whose disease has already progressed on first-line R/M therapy, treatment options such as cetuximab plus a taxane, which capitalize on the combinative ability of the 2 agents, can be considered. Notably, it is as of yet unknown what second-line treatments may be suitable to follow a checkpoint inhibitor-based first-line therapy.

## Introduction

Cancer of the head and neck accounts for >550,000 new cases annually worldwide (1, 2). About 90% of all head and neck cancers are squamous cell carcinomas (SCCs) (3). The prognosis for patients with recurrent and/or metastatic SCC of the head and neck (R/M SCCHN) is poor, with median overall survival (OS) of <1 year (4). Platinum-based combination chemotherapy had historically been the standard first-line treatment for R/M SCCHN until, in 2008, the phase 3 EXTREME trial demonstrated that the addition of cetuximab to cisplatin/carboplatin and 5-fluorouracil (5-FU), followed by cetuximab maintenance treatment, significantly improved patient outcomes compared with chemotherapy alone (5). This combination treatment increased the overall response rate (ORR) from 20 to 36%, prolonged median progression-free survival (PFS) from 3.3 to 5.6 months, and extended median OS from 7.4 to 10.1 months compared with chemotherapy alone (5). The EXTREME regimen is currently a standard first-line treatment option for patients with R/M SCCHN, as supported by international guidelines (6). However, the current treatment landscape and continuum of care are changing in light of recent data regarding pembrolizumab in certain subpopulations of R/M SCCHN (7, 8). Additionally, multiple first-line strategies are under investigation, aiming to improve outcomes for all populations with R/M SCCHN.

In this article, we outline the available evidence, as well as ongoing studies, for combining cetuximab and taxane-based chemotherapy for patients with R/M SCCHN and discuss the implications of the known safety and efficacy findings of each. We performed a thorough review of the literature to understand the mode of action of taxanes and identify key published phase 2–3 clinical trials as well as retrospective studies evaluating paclitaxel- or docetaxel-based chemotherapy in R/M SCCHN and compared their results to those of studies that investigated the current standard of care. Furthermore, we searched for ongoing phase 2–3 clinical trials on ClinicalTrials.gov and reviewed the recommendations for R/M SCCHN in the clinical guidelines from the National Comprehensive Cancer Network, the Spanish Society of Medical Oncology, and the joint European Head and Neck Society, European Society for Medical Oncology, and European Society for Radiotherapy and Oncology (6, 9, 10).

## Biological Rationale for Combining Taxanes and Platinum With Cetuximab

Designing combination regimens allows one to consider molecular mechanisms and select drugs with the potential to synergize with each other as well as to avoid the cumulative effects of their main toxicities. Indeed, cetuximab, taxanes, and platinum are appealing combination partners, as they may act synergistically to induce maximal antitumor activity. Specifically, cetuximab promotes cell cycle arrest and activation of proapoptotic molecules (11), taxanes inhibit microtubule disassembly (12), and platinum agents form DNA adducts (13, 14), all leading to apoptosis. These properties suggest that the 3 agents combined may have highly additive proapoptotic and antitumor growth effects via different molecular pathways, thereby attacking more targets within a heterogeneous tumor (15). Indeed, several preclinical studies of other cancer models have demonstrated synergistic activity between cetuximab and taxanes or taxane-like cytotoxic agents in mice as measured by cell-kill metrics and tumor growth prevention (16). Furthermore, preclinical models suggest that the antitumor activity of cetuximab can synergize with platinum-mediated DNA damage activity (11, 17), making cetuximab a suitable combination partner for either cisplatin or carboplatin. Finally, across cancer cell lines, cetuximab has demonstrated additive cell cycle arrest and killing activity with cisplatin, carboplatin, oxaliplatin, docetaxel, and paclitaxel when administered sequentially after the chemotherapy agent (18). Overall, based on observations in an array of preclinical models of various tumor types, proapoptotic synergy observed between cetuximab and the individual chemotherapy agents is widespread and may apply to multiple indications.

Another important mechanism by which cetuximab and taxane agents may cooperate in increased tumor cell killing is via their immunostimulatory effects. Cetuximab elicits various immunogenic actions in the intratumoral space, including natural killer cell–driven antibody-dependent cell-mediated cytotoxicity, cytotoxic T-cell recruitment to the tumor, and dendritic cell maturation (19), which are all thought to contribute to the antitumor activity of the drug in SCCHN. Because of these activities, cetuximab is considered a suitable combination partner for immunostimulatory chemotherapy and other immunostimulatory agents (20, 21). The available evidence from several exploratory and preclinical studies on the interaction between taxanes and various immune processes is of high interest and suggests that paclitaxel and docetaxel could cooperate with cetuximab's activity in this respect too. For example, patients with breast cancer who had responses with docetaxel or paclitaxel treatment had increased immune activity markers, including interleukin 6, and increased natural killer and lymphokine-activated killer cell activity (22). Docetaxel modulates cytotoxic T-cell, natural killer cell, and regulatory T-cell populations in non-tumor-bearing mice (23), while paclitaxel enhances dendritic cell maturation (24) and other immune actions (25). Finally, encouraging early results from preclinical and small clinical studies on the combination of taxanes and vaccines suggest that taxanes can have additive immunogenic effects with other immunostimulatory anticancer therapies (23). Therefore, there is a strong biological and mechanistic rationale behind combining cetuximab with a platinum and a taxane agent when treating patients with R/M SCCHN.

## The Continuum of Care in R/M SCCHN

The EXTREME regimen is a standard first-line regimen option for fit patients with R/M SCCHN recommended by international guidelines on the basis of the phase 3 EXTREME trial, published in 2008 (4–6). This trial compared the efficacy and safety of cetuximab plus cisplatin/carboplatin plus 5-FU for up to 6 cycles, followed by cetuximab maintenance until disease progression in the first-line treatment of R/M SCCHN. The addition of cetuximab to chemotherapy improved median PFS by 2.3 months and median OS by nearly 3 months (to 5.6 and 10.1 months, respectively); ORR was also significantly increased from 20% with chemotherapy alone to 36% with the EXTREME regimen. The safety profile of the EXTREME regimen was consistent with that expected for the administered agents, including cardiac events (associated with 5-FU), anorexia, and skin reactions, except for an increase in the number of patients who developed sepsis (9 of 219 in the EXTREME arm vs. 1 of 215 in the chemotherapy-only arm) (5). The safety and efficacy of the EXTREME regimen have been consistent in the real world, as evidenced by multiple prospective, observational studies, including DIRECT, ENCORE, and SOCCER (26–28). Therefore, the EXTREME regimen is feasible for use in everyday clinical practice.

Ongoing checkpoint inhibitor studies are reshaping the treatment landscape for specific patient subgroups with R/M SCCHN in the near future. For example, the randomized phase 3 KEYNOTE-048 study evaluates first-line treatment with pembrolizumab compared with the EXTREME regimen. In this study, patients were randomized to receive pembrolizumab monotherapy, pembrolizumab plus chemotherapy (cisplatin/carboplatin plus 5-FU), or the EXTREME regimen. Although no improvement in PFS was demonstrated and a low ORR was observed, pembrolizumab monotherapy demonstrated superior median OS in the programmed cell death ligand 1 (PD-L1) combined positive score (CPS) ≥ 20 population (14.9 months vs. 10.7 months) compared with the EXTREME regimen in patients with R/M SCCHN (7). The OS difference in the overall population showed pembrolizumab to be non-inferior to EXTREME (8). Pembrolizumab plus chemotherapy demonstrated longer OS than EXTREME (median OS, 13.0 vs. 10.7 months) (7). The OS differences in patients with CPS ≥ 20 (14.7 vs. 11.0 months) and CPS ≥ 1 (13.6 vs. 10.4 months) were significant. Patients with CPS 1-19 in the pembrolizumab monotherapy arm had a median OS of 10.8 months (HR, 0.90 [95% CI: 0.68, 1.18]). These data suggest that the OS benefit in the KEYNOTE-048 study was driven by the patients with the highest PD-L1 expression (7, 8, 29). As a result, pembrolizumab with or without chemotherapy is likely to become the first-line standard of care for patients with PD-L1–high (CPS ≥ 20) R/M SCCHN. In contrast, the benefit of pembrolizumab therapy in patients with PD-L1–low (CPS 1–20) or PD-L1–negative (CPS < 1) R/M SCCHN has not been demonstrated; therefore, the EXTREME regimen remains a standard treatment option in these patients. Of note, however, pembrolizumab has now been approved by the US Food and Drug Administration for first-line treatment of patients with R/M SCCHN as monotherapy for patients whose tumors express PD-L1 (CPS ≥ 1) or in combination with platinum and 5-FU regardless of PD-L1 expression (29).

Patients whose disease progresses on the EXTREME regimen are then eligible for second-line therapy, including but not limited to single-agent chemotherapy (e.g., taxanes) (9), a combination treatment (e.g., chemotherapy doublet), or, in light of recent advances, checkpoint inhibitor monotherapy (6, 30–32). Immune checkpoint inhibitors improve OS compared with investigator's choice of monotherapy (cetuximab, methotrexate, docetaxel) in the second- or later-line setting and in platinum-refractory patients (patients who progressed within 6 months of the last dose of platinum in the locally advanced [LA] SCCHN setting), but the ORR remains low (13.3% with nivolumab and 14.6% with pembrolizumab) (31, 33).

Taxanes can help expand the treatment options available in the first line, where they can replace 5-FU in the EXTREME regimen, as well as in second- or later-line settings and in settings where cisplatin/carboplatin treatment is unsuitable (9), where patients can receive cetuximab in combination with single-agent taxane instead. Furthermore, cetuximab-based therapy, e.g., in combination with taxanes, may become a standard second- or later-line treatment option after disease progression on pembrolizumab monotherapy. Finally, the combination of taxanes with checkpoint inhibitors is also currently being evaluated, such as in the single-arm, phase 1/2 PemDoc II study of pembrolizumab plus docetaxel (34).

## Efficacy of Cetuximab in Combination With Platinum and a Taxane for First-Line R/M SCCHN

The combination of cetuximab with platinum and a taxane has become of interest for the first-line treatment of patients with R/M SCCHN in recent years, ever since the GORTEC 2008-03 trial suggested that these combinations may yield improved ORRs and favorable OS compared with the EXTREME regimen (5, 35). Results from the large randomized TPExtreme trial of first-line cetuximab plus platinum and a taxane vs. the EXTREME regimen have been reported and confirm earlier evidence from smaller randomized or single-arm studies that has suggested that the use of first-line docetaxel or paclitaxel instead of 5-FU in the EXTREME regimen retains the regimen's good efficacy and favorable safety profile in patients with R/M SCCHN (36).

The trials with available, published data are outlined in Table 1. There were some notable differences in the taxane selected, the chemotherapy dosage, and the administration schedule of maintenance cetuximab between trials. Whereas docetaxel was selected for the phase 2 GORTEC 2008-03 study and for TPExtreme, paclitaxel was used in CSPOR-HN02, CET-INT (B490), and CETMET, and nab-paclitaxel was used in the phase 2, single-arm CACTUX trial (37–42). Furthermore, whereas paclitaxel was administered at 175 mg/m^2^ every 3 weeks (q3w) in the B490 and CETMET studies, CSPOR-HN02 used a split-dose of paclitaxel (100 mg/m^2^ on days 1 and 8 q3w) (38–40, 42, 43). Notably, treatment in the Japanese phase 2 CSPOR-HN02 study could be administered in the outpatient clinic, thus reducing time spent in the hospital and, among other things, providing more time for patients to spend with family (39, 40).

**Table 1:** Efficacy of cetuximab + platinum + taxane regimens in first-line R/M SCCHN studies.

**Study**	**No. of Patients**	**Regimen**	**ORR, %**	**PFS, months**	**OS, months**
GORTEC 2008-03 (TPEx) (35)	54	Cetuximab (400 mg/m^2^ → 250 mg/m^2^ qw) + cisplatin (75 mg/m^2^ q3w) + docetaxel (75 mg/m^2^ q3w) + G-CSF (150 μg/m^2^ q3w) *Maintenance:* cetuximab (500 mg/m^2^ q2w)	51.9	6.2	14.0
CET-INT (B490) (37, 38)	201	Cetuximab (400 mg/m^2^ → 250 mg/m^2^ qw) + cisplatin (100 mg/m^2^ q3w) (*n* = 100) *Maintenance:* cetuximab (250 mg/m^2^ qw) vs. Cetuximab (400 mg/m^2^ → 250 mg/m^2^) + cisplatin (75 mg/m^2^ q3w) + paclitaxel (175 mg/m^2^ q3w) (*n* = 91) *Maintenance:* cetuximab (250 mg/m^2^ qw)	41.8 vs. 51.7	6.0 vs. 7.0	13.0 vs. 11.0
CSPOR-HN02 (39, 40)	45	Cetuximab (400 mg/m^2^ → 250 mg/m^2^ qw)^a^ + carboplatin (AUC 2.5 on days 1 and 8 q3w, up to 6 cycles) + paclitaxel (100 mg/m^2^ on days 1 and 8) *Maintenance:* cetuximab (250 mg/m^2^ qw)	40.0	5.2	14.7
NCT02270814 (CACTUX) (41)	32	Cetuximab (400 mg/m^2^ → 250 mg/m^2^ qw) + cisplatin/carboplatin (75 mg/m^2^ q3w/AUC 5, respectively) + nab-paclitaxel (100 mg/m^2^ qw) *Maintenance:* cetuximab (250 mg/m^2^ qw) + nab-paclitaxel (100 mg/m^2^ qw)	63.0^b^	6.8^b^	18.8^b^
NCT01830556 (CETMET) (42)	85	Cetuximab (400 mg/m^2^ → 250 mg/m^2^ qw) + carboplatin (AUC 5) + paclitaxel (175 mg/m^2^ q3w) *Maintenance:* cetuximab (500 mg/m^2^ q2w) vs. Cetuximab (400 mg/m^2^ → 250 mg/m^2^ qw) + cisplatin/carboplatin (100 mg/m^2^ q3w/AUC 5, respectively) + 5-FU (1,000 mg/m^2^/24 h for 96-h continuous infusions) *Maintenance:* cetuximab (500 mg/m^2^ q2w)	51.2 vs. 47.6	6.5 vs. 4.4	10.2 vs. 8.4

*5-FU, 5-fluorouracil; AUC, area under the curve; CACTUX, Cisplatin, nab-Paclitaxel, and Cetuximab; CSPOR, Comprehensive Support Project for Oncology Research; G-CSF, granulocyte colony-stimulating factor; GORTEC, Groupe d'Oncologie Radiothérapie Tête Et Cou; nab-paclitaxel, albumin-bound paclitaxel; NCT, ClinicalTrials.gov identifier; ORR, overall response rate; OS, overall survival; PFS, progression-free survival; q2w, every 2 weeks; q3w, every 3 weeks; qw, once weekly; R/M SCCHN, recurrent and/or metastatic squamous cell carcinoma of the head and neck; TPEx, cisplatin, docetaxel, and cetuximab*.

a *Cetuximab qw approved in Japan until disease progression or unacceptable toxicities*.

b *Results from a planned interim analysis of the CACTUX trial*.

The patient populations treated with a regimen consisting of cetuximab, a platinum agent, and a taxane in the TPExtreme, GORTEC 2008-03, B490, and CSPOR-HN02 studies were somewhat comparable to the patient population of the EXTREME study, although it is difficult to make any solid conclusions given the many different prognostic factors associated with drug efficacy (e.g., in-field lesions vs. metastatic only, Eastern Cooperative Oncology Group performance status [ECOG PS], carboplatin vs. cisplatin) (5, 35, 37–40). Notably, a higher percentage of patients in the GORTEC 2008–03 (70.4%) and CSPOR-HN02 (82.2%) studies had metastatic disease compared with patients in the EXTREME study (47%) (35, 39, 40). By contrast, 50.5% of the population enrolled in the cetuximab, platinum, and paclitaxel arm of the B490 study had some form of metastatic disease, and only 58% of patients in the CETMET study had metastatic disease (37, 38, 42). Furthermore, unlike EXTREME, the TPExtreme, GORTEC 2008-03, B490, and CSPOR-HN02 studies all exclusively enrolled patients with an ECOG PS of 0 or 1 (5, 35, 37–40). Finally, in the B490 study, 4.4% of patients in the cetuximab, platinum, and paclitaxel arm had previously received cetuximab treatment in the LA setting (37, 38). In agreement with the literature, human papillomavirus status was prognostic in all studies that tested for it and had patient samples large enough to draw conclusions on this topic.

The GORTEC 2008-03, B490, CETMET, and CSPOR-HN02 studies all met their primary endpoints. In these studies, clinical efficacy of the treatment of patients with first-line R/M SCCHN did not appear to be compromised upon the substitution of 5-FU with a taxane in the EXTREME regimen (Table 1): Median PFS ranged from 5.2 to 7.0 months, which is in alignment with the median PFS observed in the EXTREME phase 3 trial (5.6 months) (5, 35, 37–40, 42). Survival was also consistent between trials, with median OS ranging from 10.2 to 14.7 months (5, 35, 37–40, 42). Finally, response rates may potentially be higher with this modified regimen: ORRs ranged from 40.0 to 51.7% when 5-FU was replaced with a taxane in the EXTREME regimen (5, 35, 37–40).

The topline findings from TPExtreme were presented at the 2019 American Society of Clinical Oncology Annual Meeting (36). Median OS for the TPEx vs. EXTREME arms was not significantly different (14.5 vs. 13.4 months; HR, 0.87 [95% CI, 0.71–1.05]; *p* = 0.15); but the OS in the EXTREME arm was unexpectedly higher than historical randomized data, leading to a decrease in the trial power (36, 44). However, the TPEx regimen was significantly better tolerated than the EXTREME regimen.

In summary, although treatment regimens differ by selection of taxane and sometimes dose, the survival and safety results appear consistent from study to study. While the survival, response, and manageable safety profile of the regimens studied in the GORTEC 2008-03, B490, CETMET, and CSPOR-HN02 studies are promising, we eagerly anticipate the full published results of the ongoing European randomized TPExtreme trial, which directly compares the TPEx and EXTREME regimens, within the coming year (44). A detailed list of ongoing studies is provided in Table 2.

**Table 2:** Ongoing studies of cetuximab + platinum + taxane regimens in first-line R/M SCCHN.

**Study**	**Estimated enrollment**	**Regimen**	**Primary endpoint**	**Results expected/presented**
NCT02268695 (TPExtreme; randomized trial)	*N* = 540 1L R/M SCCHN^a^ *Countries:* France Germany Spain	Cetuximab (400 mg/m^2^ → 250 mg/m^2^ qw) + cisplatin (75 mg/m^2^ q3w) + docetaxel (75 mg/m^2^ qw) + G-CSF (150 μg/m^2^ q3w) *Maintenance:* cetuximab (500 mg/m^2^ q2w) vs. Cetuximab (400 mg/m^2^ → 250 mg/m^2^ qw) + cisplatin (100 mg/m^2^ q3w) + 5-FU (1000 mg/m^2^/24 h for 96-h continuous infusions) *Maintenance:* cetuximab (250 mg/m^2^ qw)	OS	December 2018/ preliminary results presented June 2019
NCT02124707 (phase 2)	*N* = 38 1L R/M SCCHN^a^^,^^b^ *Countries:* United States	Cetuximab (400 mg/m^2^ → 250 mg/m^2^ qw) + carboplatin (AUC 2) + paclitaxel (135 mg/m^2^ qw) *Maintenance:* cetuximab (250 mg/m^2^ qw)	OS	February 2020
NCT02270814 (CACTUX; phase 2) (41)	*N* = 70 1L R/M SCCHN^c^ *Countries:* United States	Cetuximab (400 mg/m^2^ → 250 mg/m^2^ qw) + cisplatin/carboplatin (75 mg/m^2^ q3w/AUC 5, respectively) + nab-paclitaxel (100 mg/m^2^ qw) *Maintenance:* cetuximab (250 mg/m^2^ qw) + nab-paclitaxel (100 mg/m^2^ qw)	PFS	May 2021
NCT01437449 (TPC regimen; phase 2)	*N* = 27 1L R/M SCCHN^c^ *Countries:* United States	Cetuximab (400 mg/m^2^ → 250 mg/m^2^ qw) + cisplatin (30 mg/m^2^ qw) + docetaxel (30 mg/m^2^ qw) *Maintenance:* Unspecified	ORR	January 2021
UMIN000015405^d^ (TEMPER study; phase 2)	*N* = 180 1L R/M SCCHN^a^	Cetuximab + carboplatin + docetaxel vs. Cetuximab + cisplatin + 5-FU		N/A

*1L, first-line; 5-FU, 5-fluorouracil; AUC, area under the curve; CACTUX, Cisplatin, nab-Paclitaxel, and Cetuximab; G-CSF, granulocyte colony-stimulating factor; LA, locally advanced; nab-paclitaxel, albumin-bound paclitaxel; N/A, not available; NCT, ClinicalTrials.gov identifier; ORR, overall response rate; OS, overall survival; PFS, progression-free survival; q3w, every 3 weeks; qw, once weekly; R/M SCCHN, recurrent and/or metastatic squamous cell carcinoma of the head and neck; TPC, docetaxel, cisplatin, and cetuximab; TPEx, cisplatin, docetaxel, and cetuximab*.

a *Either previously untreated or progressed on therapy in the LA setting 6+ months ago*.

b *History of prior cumulative exposure to >300 mg/m^2^ cisplatin, AUC of 18 of carboplatin, or their combined equivalent within 1 year prior to enrollment was an exclusion criterion*.

c *Either previously untreated or completed therapy in the LA setting 3–4+ months ago*.

d *Dosing information not available*.

## Safety and Administration Differences Between Taxanes and 5-FU in Combination With Cetuximab

As discussed, the combination of cetuximab, platinum, and a taxane has been shown in smaller studies to have survival comparable to that of the EXTREME regimen. Importantly, as 5-FU and taxanes have distinct associated toxicities, they can be used to treat different patient subgroups with R/M SCCHN with specific contraindications to chemotherapy agents while maintaining optimal efficacy. Indeed, observational studies have showed that physicians do not include 5-FU in 14–45% of therapeutic plans, indicating that 5-FU is not systematically prescribed as part of the EXTREME regimen, likely due to differences in its safety profile and administration (26, 27). The TPExtreme study results showed that replacing 5-FU with taxanes led to a better safety profile.

### Safety Profiles

5-FU has been associated with a high risk of severe toxicity in patients with dihydropyrimidine dehydrogenase [DPD; the enzyme that catabolizes 5-FU (45)] deficiency (3–5% occurrence rate in the general population) (46) and is therefore contraindicated in this patient population. Therefore, patients with DPD deficiency should instead be redirected to receive a taxane as a substitute.

Grade ≥ 3 cardiac toxicity was reported in the EXTREME trial in 7% of patients (5), which is consistent with the known cardiac toxicity of 5-FU. Although some cardiac events can be associated with taxanes as well (discussed below), the panel of cardiac adverse events (AEs) differs between 5-FU and taxanes. The most commonly described cardiotoxic effects of 5-FU are angina and ischemic events, with a frequency of ≤ 68% in the literature (47). A literature review spanning the years 1969 to 2007 including 377 patients treated with 5-FU reported the frequency of specific cardiotoxic events, including myocardial infarction (22%) and arrhythmias (23%) (48). Instances of sudden death and thromboembolism have also been reported in association with high-dose infusional 5-FU (49). Finally, risk of hypotension secondary to cardiological complications, especially in patients living in geographic regions with hot climates, could prevent effective renal clearance of the cisplatin component included in the treatment regimen, potentially exacerbating cisplatin-associated toxicities in parallel. In contrast, the most common cardiac event associated with taxane treatment is arrhythmia, and more specifically, bradyarrhythmia, which has been reported in <0.1–31% of patients receiving paclitaxel. Ischemia has been reported in <1–5% of patients receiving paclitaxel and 1.7% of those receiving docetaxel (47, 50, 51). Finally, no documented rate of thromboembolic events with either docetaxel or paclitaxel has been published (52). Because the majority of cardiac events secondary to chemotherapy treatment are unpredictable and potentially fatal, cardiac toxicity is an important consideration for patient selection when prescribing chemotherapy regimens.

Taxanes are associated with a different set of characteristic AEs. Hypersensitivity reactions, a known AE with taxanes, have been reported to occur in approximately 10% of patients (53). Therefore, steroids are required to reduce allergic reactions when administering taxanes (54, 55). In addition, depending on patient age and the regimen combination itself, administration of granulocyte colony-stimulating factor may be required to reduce the risk of neutropenia (54, 55). Indeed, the B490 and CSPOR-HN02 studies noted higher rates of grade 3–4 non-febrile neutropenia (42% [grade ≥ 3] and 68%, respectively), compared with those observed in EXTREME in both treatment arms (22–23%) (5, 37–40). Additionally, dependent upon the dose administered, the schedule of administration, and the duration of the infusion, neurotoxicity has been reported with paclitaxel (56). Thus, patients with preexisting neurological conditions may be better suited to receive 5-FU.

A detailed list of AEs in the GORTEC 2008-03, B490, CETMET, and CSPOR-HN02 studies is presented in Table 3. Although tolerance and compliance were significantly improved with the TPEx regimen compared with EXTREME, full safety results of this trial have not yet been published (36).

**Table 3:** Safety profile of cetuximab + platinum + taxane: grade ≥ 3 AEs occurring in ≥ 5% of patients in the cetuximab + platinum + taxane arm.

	**Incidence in each study, %**		
**AE**	**GORTEC 2008–03 (TPEx)** **(35)** **(*N* = 54)**	**CET-INT (B490)** **(37, 38)** **(*n* = 91)**	**CSPOR-HN02** **(39, 40)** **(*N* = 47)**
**Total**	NR	72.5	NR
**Hematologic events**			
Non-febrile	20.4	41.8	68.1
neutropenia			
Febrile neutropenia	7.4	6.6	8.5
Leukopenia	3.7	29.7	NR
Lymphopenia	3.7	5.5	NR
Anemia	3.7	6.6	6.4
**Non-hematologic events**			
Fever/infection	7.4	8.8	NR
Hyponatremia and	5.6	2.2, 4.4	4.3
hypokalemia			
Hypersensitivity	5.6	NR	NR
Oral mucositis	3.7	4.4	2.1
Asthenia/fatigue	3.7	26.4	NR
Anorexia	3.7	0.0	6.4
**Skin toxicities**	16.7		
Skin rash	NR	15.4	NR
Skin reaction	NR	NR	14.9

*AE, adverse events; CSPOR, Comprehensive Support Project for Oncology Research; GORTEC, Groupe d'Oncologie Radiothérapie Tête Et Cou; NR, not reported*.

### Administration Differences

In addition to known differences in their safety profiles, taxanes and 5-FU require different considerations in terms of administration as well as prophylactic and reactive AE management. Whereas a taxane can be administered in short infusions (3 h; paclitaxel, 175 mg/m^2^ q3w; docetaxel, 75 mg/m^2^ q3w), 5-FU requires a 4- to 5-day continuous infusion (1,000 mg/m^2^/d) in every chemotherapy cycle. Furthermore, the cisplatin dose used in the EXTREME regimen (100 mg/m^2^ q3w) is higher compared with that used in combination with taxanes (75 mg/m^2^ q3w) (5, 37, 38). Historically, patients have been hospitalized for the duration of the 5-FU infusion (resulting in increased costs), and this was highly inconvenient for both the patient and the institution (39). More recently, subcutaneously implanted “port” catheters and portable pumps have become an option allowing administration of 5-FU in the outpatient setting; however, the pumps require a peripherally inserted central venous line, which in turn can put the patient at risk for complications during use (≈ 25% of cases) or be associated with failure of line removal (≈ 15% of cases) as well as infections of the exit site and bloodstream (at a rate of ≈ 3 and ≈ 2%, respectively) (57). Indeed, most patients continue to require hospitalization for continuous infusion of 5-FU, leading to decreased quality of life for the patient via less time spent with family and inconveniences due to the availability of access to hospital facilities. By contrast, patients using portable pumps still have to return to the hospital at the end of the 4-day infusion. This can be viewed as either an inconvenience to the patient because of an additional hospital visit or as an advantage because the patient can be examined for any issues (e.g., renal insufficiency) by a specialized practitioner, thus reducing the risk of undetected problems.

In summary, although the EXTREME regimen is a longstanding first-line treatment option for R/M SCCHN, patients with preexisting cardiac problems, DPD deficiencies, and other risk factors may be more suited to receive a taxane than 5-FU during first-line combination therapy. Indeed, optimizing the first-line management of R/M SCCHN via appropriate patient selection is not only important for the patient's quality of life, but it is also necessary to help maintain the patient's ECOG PS and fitness upon disease progression to allow for additional therapies in the second- and later-line settings.

## Uses of Cetuximab Plus a Taxane

Patients whose disease has progressed on the EXTREME regimen or who have platinum-refractory disease have several remaining treatment options, including single-agent chemotherapy (docetaxel, methotrexate) and checkpoint inhibitor therapy (nivolumab, pembrolizumab). For patients with PD-L1–high tumors whose disease progresses on first-line pembrolizumab monotherapy, the EXTREME regimen or TPEx regimen will likely become a second-line treatment option. As cetuximab plus either docetaxel or paclitaxel has shown efficacy as second- or later-line treatment in patients with R/M SCCHN, this combination may be a suitable treatment option for patients with disease progression on first-line pembrolizumab plus platinum-based chemotherapy.

### Cisplatin/Carboplatin-Unsuitable or Poor PS R/M SCCHN

Patients entering or progressing in the R/M SCCHN continuum of care for whom a platinum-based chemotherapy regimen is not suitable due to a poor ECOG PS, contraindication, or too-short time to recurrence since the last dose of platinum (e.g., treatment-free interval <6 months) are not eligible to receive the EXTREME regimen and thus require an alternative treatment approach. A cetuximab-taxane regimen could confer benefit in this patient population. Indeed, the combination of cetuximab plus docetaxel or paclitaxel has been shown to have a manageable safety profile and promising efficacy in various prospective and retrospective studies of patients with either first-line or platinum-refractory R/M SCCHN (Table 4) (2, 58–60). Although these studies had small numbers of enrolled patients and were single arm or retrospective, the results suggested that patient selection for cetuximab-plus-taxane combinations in the settings of cisplatin/carboplatin unsuitability or poor PS may result in high ORRs and disease control in certain poor-prognosis populations. Currently, no further investigations of the combination of cetuximab plus a taxane in patients who are unable to receive cisplatin, carboplatin, or an aggressive chemotherapy regimen are being conducted.

**Table 4:** Studies of cetuximab + taxane–only regimens.

**Study**	**No. of patients, setting**	**Regimen**	**ORR, %**	**PFS, months**	**OS, months**
**PROSPECTIVE**					
Hitt et al. (58); phase 2	*n* = 46 1L R/M SCCHN*^a^*	Cetuximab (400 mg/m^2^ → 250 mg/m^2^ qw) + paclitaxel (80 mg/m^2^ qw) until progression or unacceptable toxicity	54	4.2	8.1
Knoedler et al. (2); phase 2	*n* = 84 All patients had prior platinum therapy but no prior cetuximab	Cetuximab (400 mg/m^2^ → 250 mg/m^2^ qw) + docetaxel (35 mg/m^2^ qw) *Maintenance:* cetuximab 250 mg/m^2^ qw	11	3.1	6.7
**RETROSPECTIVE**					
Posch et al. (59)	*n* = 31 1L R/M SCCHN, unselected	Cetuximab (500 mg/m^2^) + docetaxel (50 mg/m^2^) q2w	13	4.0	8.3
Bernad et al. (60)	*n* = 148 Mostly unfit for aggressive therapy (including EXTREME)	Cetuximab (400 mg/m^2^ → 250 mg/m^2^ qw) + paclitaxel (80 mg/m^2^ qw)	47	7	10

*1L, first-line; LA, locally advanced; ORR, overall response rate; OS, overall survival; PFS, progression-free survival; qw, once weekly; R/M SCCHN, recurrent and/or metastatic squamous cell carcinoma of the head and neck*.

a *Either previously untreated or progressed on therapy in the LA setting 6+ months ago*.

### Later-Line R/M SCCHN

Some single-center or retrospective studies have provided evidence for the use of cetuximab plus a taxane in later-line R/M SCCHN. Unfortunately, these studies enrolled patients in various lines of treatment, with patients with R/M SCCHN in cisplatin/carboplatin-unsuitable, second-, and later-line settings all receiving the same treatment and analyzed as a pooled data set, thereby confounding interpretation of survival outcomes between trials and regimens. A single-arm trial of 59 patients receiving cetuximab and paclitaxel, of whom 75% were previously untreated in the R/M setting, yielded an ORR of 47.5% and median PFS and OS of 7.7 months and 13.2 months, respectively (61). Sosa et al reported an ORR, median PFS, and median OS of 55%, 4.0 months, and 10.0 months, respectively, in 33 patients in whom first-line platinum-based therapy had failed (62). Similarly, Jimenez et al described a study of 22 patients with second-line or cisplatin/carboplatin-unsuitable R/M disease who were treated with cetuximab and paclitaxel and achieved an ORR of 55% (95% CI, 31–76%), a median PFS of 5.4 months, and a median OS of 9.1 months (63). Finally, Peron et al reported an ORR of 38%, median PFS of 3.9 months, and OS of 7.6 months in a cohort of 42 patients with second-line R/M SCCHN (64). Taken together, the results of the available retrospective studies of cetuximab-plus-taxane regimens align with what has been observed in single-arm and randomized, prospective trials, seemingly independent of mixed cisplatin/carboplatin-unsuitable, second- or later-line settings. Although findings still need to be validated in the randomized, prospective setting, the combination of cetuximab and a taxane may be a viable treatment option for patients with platinum-refractory or second-line disease. Additionally, follow-up therapies after patients progress on checkpoint inhibitor-based treatment remain to be adequately investigated.

## Conclusions

The substitution of 5-FU with a taxane is being investigated as a potential method to further improve survival and response rates in first-line R/M SCCHN. Such combination regimens can take advantage of the ability of cetuximab to synergize antitumor activity with platinum and taxane agents, as demonstrated in preclinical studies. Available evidence suggests that cetuximab plus platinum and a taxane is a more favorable combination with efficacy similar to that of the EXTREME regimen and a favorable safety profile in patients with R/M SCCHN. Indeed, it appears that this combination may further improve on the ORR of the EXTREME regimen with similar survival outcomes (36). The landscape in R/M SCCHN is evolving, and the first-line standard of care is becoming fragmented due to newly available treatment options, e.g., for the population with PD-L1–high disease. Other patients, such as those with PD-L1–low and PD-L1–negative tumors and those with high tumor burden, are more likely to benefit from cetuximab plus platinum-based chemotherapy. The TPEx regimen is a first-line treatment option for patients with R/M SCCHN. For patients with a high risk of toxicity with 5-FU, TPEx and related regimens present an interesting treatment approach with efficacy similar to that of the EXTREME regimen. Additionally, patients unable to receive cisplatin- or carboplatin-based chemotherapy, or those with poor prognosis or later-line R/M SCCHN, are suitable candidates for cetuximab plus paclitaxel or docetaxel treatment as well as other available therapy options (e.g., checkpoint inhibitors, single-agent chemotherapy).

## Author Contributions

All authors contributed equally to the conception of the intellectual content, interpretation of the data, and writing of the manuscript. All authors also reviewed any revisions that were made and provided their final approval of the manuscript.

### Conflict of Interest Statement

JG has served on advisory boards for AstraZeneca, Bristol-Myers Squibb, Innate Pharma, and Merck Healthcare KGaA and has received grants for research from GSK, Bristol-Myers Squibb, Chugai, and Merck Healthcare KGaA. MT has received honoraria from Bayer, Bristol-Myers Squibb, Eisai, Merck Serono, and Otsuka, and served as a consultant or advisor to Bayer, Merck Sharp & Dohme Pharmaceuticals, Ono Pharmaceutical, and Pfizer. MT has also received research funding from AstraZeneca, Bayer, Boehringer Ingelheim, Eisai, Merck Sharp & Dohme, Novartis, NanoCarrier, Ono Pharmaceutical, and Pfizer. LL has served as a consultant or advisor for and received research funding from Astra-Zeneca, Bayer, Boehringer-Ingelheim, Bristol-Myers Squibb, Debiopharm, Eisai, Merck Serono, Merck Sharp & Dohme Pharmaceuticals, Novartis, Roche, and Sobi. LL has also received travel compensation from Bayer, Debiopharm, Merck Serono, and Sobi. UK has served as a consultant or advisor for Bristol-Myers Squibb, Merck Serono, AstraZeneca, Merck Sharp & Dohme Oncology, Pfizer, served on a Speakers' Bureau with Merck Sharp & Dohme Oncology, Bristol-Myers Squibb, Novartis, Merck Serono, Glycotope GmbH, AstraZeneca, received reimbursement for travel, accommodations, and expenses from AstraZeneca, Merck Serono, Merck Sharp & Dohme Oncology, Ipsen, received honoraria from Bristol-Myers Squibb, Merck Healthcare KGaA, Merck Sharp & Dohme Oncology, AstraZeneca, Novartis, Pfizer, Glycotope GmbH, Roche/Genentech and received institutional research funding from Pfizer and AstraZeneca/MedImmune. SF has no relevant disclosures. PW is an employee of Merck Healthcare KGaA. RM is a consultant/advisor with honoraria from Merck Healthcare KGaA, Merck Sharp & Dohme, and AstraZeneca.

## Abbreviations

1L: first-line
5-FU: 5-fluorouracil
AE: adverse event
AUC: area under the curve
CACTUX, Cisplatin, nab-Paclitaxel: and Cetuximab
CSPOR: Comprehensive Support Project for Oncology Research
DPD: dihydropyrimidine dehydrogenase
ECOG PS: Eastern Cooperative Oncology Group performance status
EXTREME: cetuximab plus cisplatin/carboplatin plus 5-fluorouracil followed by maintenance cetuximab
GORTEC: Groupe d'Oncologie Radiothérapie Tête Et Cou
nab-paclitaxel: albumin-bound paclitaxel
NCT: ClinicalTrials.gov identifier
NR: nor reported
ORR: overall response rate
OS: overall survival
PFS: progression-free survival
q2w: every 2 weeks
q3w: every 3 weeks
qw: once weekly
R/M: recurrent and/or metastatic
SCCHN: squamous cell carcinoma of the head and neck
TPEx, cisplatin, docetaxel: and cetuximab.
